# Characterization of the chicken melanocortin 5 receptor and its potential role in regulating hepatic glucolipid metabolism

**DOI:** 10.3389/fphys.2022.917712

**Published:** 2022-10-06

**Authors:** Xiao Zhang, Jiancheng Su, Tianjiao Huang, Xinglong Wang, Chenlei Wu, Jing Li, Juan Li, Jiannan Zhang, Yajun Wang

**Affiliations:** Key Laboratory of Bio-resources and Eco-environment of Ministry of Education, Animal Disease Prevention and Food Safety Key Laboratory of Sichuan Province, College of Life Sciences, Sichuan University, Chengdu, China

**Keywords:** MC5R, MRAP1, functional analysis, tissue expression, THRSPA, ELOVL6

## Abstract

Melanocortin receptors (MC1R-MC5R) and their accessory proteins (MRAPs) are involved in a variety of physiological processes, including pigmentation, lipolysis, adrenal steroidogenesis, and immunology. However, the physiological roles of MC5R are rarely characterized in vertebrates, particularly in birds. In this work, we cloned the full-length cDNA of chicken MC5R and identified its core promoter region. Functional studies revealed that cMC5R was more sensitive to ACTH/α-MSH than β-MSH/γ-MSH, and was coupled to the cAMP/PKA signaling pathway. We demonstrated that MRAP2 decreased MC5R sensitivity to α-MSH, whereas MRAP1 did not have a similar effect, and that both MRAPs significantly reduced MC5R expression on the cell membrane surface. Transcriptome and qPCR data showed that both MRAP1 and MC5R were highly expressed in chicken liver. Additionally, we observed that ACTH might increase hepatic glucose production and decrease lipogenesis in primary hepatocytes, and dose-dependently downregulated the expression levels of *ELOVL6* and *THRSPA* genes. These findings indicated that ACTH may act directly on hepatocytes to regulate glucolipid metabolism, which will help to understand the function of MC5R in avian.

## Introduction

Melanocortin receptors (MCRs) are a set of five G-protein coupled receptors (MC1R-MC5R) that may be divided into two groups depending on ligand selectivity for α-melanocyte-stimulating hormone (α-MSH) and adrenocorticotropin (ACTH) in bony vertebrates (Cone, 2006). The MC2R, also known as the adrenocorticotropic hormone receptor (ACTHR), can only be activated by ACTH when melanocortin receptor accessory protein 1 (MRAP1) is present, while the other receptors (MC1R, MC3R, MC4R, and MC5R) can be activated by α-MSH or ACTH in the presence or absence of MRAP1/MRAP2 (Chan et al., 2009; Rouault et al., 2017b; Yang and Harmon, 2017). As a single transmembrane protein that forms a homodimer, MRAPs may interact with and regulate the trafficking and signaling of all MCRs (Chan et al., 2009). In numerous vertebrates, MCRs interact with MRAPs and play a role in pigmentation, lipolysis, adrenal steroidogenesis, energy homeostasis, immunology, and cardiovascular function (Cooray and Clark, 2011; Dores et al., 2016; Scanes and Pierzchala-Koziec, 2021; Ji et al., 2022).

In the chicken genome, there are five paralogous MCRs and two MRAPs (Ling et al., 2004; Barlock et al., 2014; Zhang et al., 2017; Thomas et al., 2018). In recent years, the pharmacological characterization and physiological activity of chicken MCRs (cMCRs) have been widely explored (Scanes and Pierzchala-Koziec, 2021). It has been shown that chicken MC1R binds to the radioligand or responds to α-MSH/ACTH by promoting intracellular cAMP accumulation (Ling et al., 2003; Ling et al., 2004; Mundy, 2005). MC1R of dark-feathered chickens with a Glu to Lys mutation at position 92 showed constitutive activity, which was associated with feather color in chickens (Ling et al., 2003). When chicken MC2R was co-expressed with MRAP1 in CHO cells, the ligand selectivity properties of the receptor were identified, demonstrating that cMC2R can be activated by ACTH_1-24_, but not by NDP-MSH (Barlock et al., 2014). Further research revealed that cMC2R was potently activated by chicken or human ACTH only in the presence of MRAP1 (Zhang et al., 2017; Thomas et al., 2018). The major melanocortin receptor expressed in the adrenal gland, MC2R, was thought to be a key regulator of the hypothalamus-pituitary-adrenal (HPA) axis in stress adaption (Dores and Garcia, 2015; Thomas et al., 2018). Chicken MC3R and MC4R were predominantly expressed in the hypothalamus, which were activated by both α-MSH and ACTH. Unlike cMC2R, their constitutive activity and ligand sensitivity were affected by both MRAP1 and MRAP2 (Zhang et al., 2017; Thomas et al., 2018). Single nucleotide polymorphisms (SNPs) in MC4R have been reported to be associated with the differences in body weight and egg production in chickens (Karim and Aggag, 2018; Kubota et al., 2019).

In addition, previous studies on MC5R in chickens have offered some information. Ling et al. investigated the ability of chicken MC5R to couple with the intracellular messenger cAMP and observed that the EC50 values with different ligands (human α-MSH and ACTH_1-24_) were equivalent (Ling et al., 2004). Furthermore, recent studies have shown that MRAP1 supplementation improved chicken MC5R sensitivity to human ACTH_1-24_, and demonstrated that the KKRRP motif of ACTH_1–24_ was needed for complete activation of cMC5R when co-expressed with MRAP1 in cultured CHO cells (Thomas et al., 2018; Dores et al., 2020). However, chicken MRAP2 had no influence on cMC5R sensitivity to human ACTH_1–24_ (Thomas et al., 2018). Another study indicated the F254A mutation in chicken MC5R displayed a significant increase in basal activity and significantly reduced the reactivity to α-MSH/NDP-α-MSH. They also found that the MC5R mutants D119A and D204A were completely unresponsive to three agonists (α-MSH, NDP-α-MSH and SHU9119), suggesting that the acidic amino acids D119 and D204 in cMC5R played an essential role in intracellular signal transduction (Min et al., 2019).

However, some key information, including the physiological role of MC5R in chickens, remains uncertain. Although human melanocortin peptides have been used to characterize chicken melanocortin receptors in previous studies. α-MSH is identical at all positions in chicken and human, but Arg15 and Ile20 in chicken ACTH differ from Lys15 and Val20 in human ACTH. Arg15 is the first amino acid of the KKRRP motif, which may play a key role in the activation of cMC5R (Dores et al., 2020). We hypothesized that the mutations in the ligand ACTH might yield different results in functional studies. In addition, MC5R has been detected to be expressed in the liver, adrenal gland, kidney, fat, and lung of chickens (Takeuchi and Takahashi, 1998; Yabuuchi et al., 2010; Thomas et al., 2018; Min et al., 2019), but its physiological functions in these tissues remain unknown. The high level of MC5R expression in chicken liver increases the possibility that the liver is an important target tissue for melanocortin (Thomas et al., 2018; Min et al., 2019). In the present study, we evaluated the effects of the accessory protein MRAPs on the sensitivity of cMC5R to natural chicken melanocortin peptides and investigated the role of ACTH on avian liver metabolism.

## Materials and methods

### Chemicals, primers, peptides, and antibodies

All chemicals were purchased from Sigma-Aldrich (St. Louis, MO, United States) and the restriction enzymes were obtained from TaKaRa (Dalian, China). Chicken (c-)ACTH_1–39_, α-MSH (acetyl-α-MSH), β-MSH, and γ-MSH were synthesized by GL Biochem Ltd (Shanghai, China). The synthesized peptides have a purity of above 95% (as determined by HPLC) and their structures have been confirmed by mass spectrometry. H89 (371,963), MDL (444,200), forskolin (344,273) and 8-Br-cAMP (203,800) were purchased from Calbiochem (Merck KGaA, Darmstadt, Germany). Antibodies for total CREB (48H2) rabbit mAb (1:1,000, #9197), phosphorylated CREB (Ser133) (87G3) rabbit mAb (1:1,000, #9198) and anti-rabbit IgG, HRP-linked antibody (1:5,000, #7074) were purchased from Cell Signaling Technology (CST, Bevely, MA). All primers used in this study were synthesized by Beijing Genome Institute (BGI, China) and listed in Supplementary Table 1.

### Animals

Adult chickens and chicks of the Lohmann Layer strain were purchased from local commercial companies. All animal experiments were carried out in accordance with the Guidelines for Experimental Animals issued by the Ministry of Science and Technology of the People’s Republic of China. All animal experimental protocols used in this study were approved by the Animal Ethics Committee of College of Life Sciences, Sichuan University.

### RNA extraction, RT-PCR, and quantitative real-time PCR assays

Three female and three male adult chickens were sacrificed and tissues including the cerebellum, midbrain, cerebrum, hindbrain, hypothalamus, anterior pituitary, kidney, liver, lung, muscle, skin, testis, adrenal gland, abdominal fat, heart, spleen, and ovary were immediately collected. Fresh tissues were frozen in liquid nitrogen and then placed in a refrigerator at −80°C until use. RNAzol reagent (Molecular Research Center, Cincinnati, OH) was used to extract total RNA, and all operations were performed in accordance with the manufacturer’s instructions. The total RNA obtained was resuspended in H_2_O treated with diethylpyrocarbonate (DEPC). These RNA samples were then reverse transcribed by Moloney murine leukemia virus (MMLV) reverse transcriptase (Takara, Dalian, China). In brief, oligodeoxythymide (0.5 μg) and total RNA (2 μg) were mixed in a total volume of 5 μl, incubated at 70°C for 10 min, and cooled at 4°C for 2 min. Then, the buffer containing 0.5 mM each of deoxynucleotide triphosphate and 100 U MMLV reverse transcriptase were added into the reaction mix, for a total volume of 10 μl. The reverse transcription (RT) reaction was performed at 42°C for 90 min.

cDNA samples were subjected to quantitative real-time PCR assay of chicken MC5R mRNA levels in different chicken tissues, as described in our previous study (Fang et al., 2021; Zhang et al., 2021). Quantitative real-time PCR (qPCR) was performed on the CFX96 Real-time PCR Detection System (Bio-Rad, Hercules, CA). Briefly, the reaction system contained 1 μl of EvaGreen (Biotium Inc., Hay-ward, CA), 1 μl of cDNA, 1 × PCR buffer, 0.2 mM each dNTP, 0.2 mM each primer, 0.5 U Taq DNA polymerase (TaKaRa) and RNase-free H_2_O to a final volume of 20 μl. The PCR profile consisted of 40 cycles of 94°C for 3 min, followed by 94°C for 15 s, 60°C for 15 s, and 72°C for 20 s. To assess the specificity of PCR amplification, melting curve analysis and agarose gel electrophoresis were performed at the end of the PCR reaction to confirm that a specific PCR band was produced. In addition, the identity of PCR products for all genes was confirmed by sequencing.

### Cloning the full-length cDNA and promoter regions of chicken MC5R

To construct the expression plasmid of MC5R for functional assay, several specific primers (Supplementary Table S1) were designed according to the chicken MC5R sequence (KF670718.1) in GenBank, and PCR was performed using these primers and liver-derived cDNA as a template to obtain the coding region sequence of cMC5R. The amplified PCR products were cloned into the pcDNA3.1 (+) vector (Invitrogen, Carlsbad, CA) and sequenced by Tsingke Company (Beijing, China).

To determine the complete gene structure of chicken MC5R, gene-specific primers (Supplementary Table S1) were used to amplify the 5′-untranslated region (5′-UTR) and 3′-UTR of chicken MC5R from the adult chicken liver using the SMART-RACE cDNA amplification Kit (Clontech, Palo Alto, CA). The amplified PCR products were cloned into the pTA2 vector (TOYOBO) and sequenced by the Tsingke Company (Beijing, China). The sequences of 5′-UTR and 3′-UTR of the chicken MC5R gene were then compared to the chicken genome database (www.ensembl.org/gallus_gallus). Finally, the full-length cDNA of MC5R was determined based on the sequences of 5′- and 3′-cDNA ends with an overlapping region, which has been deposited in GenBank with an accession number OP259502.

To determine the chicken MC5R promoter region, specific primers (Supplementary Table S1) were generated to amplify 5′-flanking regions of different lengths from the genomic DNA template. These PCR products were cloned into the pGL3-Basic vector (Promega, Madison, WI) and sequenced. In this study, the transcription start site of MC5R exon 1 was designed as “+1”, and the first nucleotide upstream of the transcription start site was designed as “−1”. Finally, a series of promoter-luciferase reporter constructs for cMC5R (P1: −1827/+171-Luc, P2: −828/+171-Luc, P3: −343/+171-Luc, P4: −66/+171-Luc) were obtained.

### Functional analysis of chicken MC5R, MRAP1 and MRAP2

The expression plasmids encoding MC5R, MRAP1 and MRAP2 were established by cloning their entire open reading frame (ORF) into the pcDNA3.1 (+) expression vector (Invitrogen). Chinese hamster ovary (CHO) cells transiently expressing MC5R were treated with chicken ACTH_1–39_/α-MSH/β-MSH/γ-MSH (10^−12^–10^−6^ M, 6 h), and the receptor-activated cAMP signaling pathway was then monitored using the pGL3-CRE-luciferase reporter system according to our previously established methods (Zhang et al., 2017; Zhang et al., 2020).

In brief, CHO cells were cultured on a six-well plate (Nunc, Roskilde, Denmark) and grown for 24 h before transfection. The cells were then transfected with a mixture containing 700 ng pGL3-CRE-Luciferase reporter construct, 200 ng of receptor expression plasmid (or empty pcDNA3.1 vector as a negative control), 20 ng of pRL-TK construct (containing a Renilla luciferase gene, used as an internal control), and 2 μl jetPRIME transfection reagent (Polyplus Transfection, Illkirch, France) in 200 μL buffer. Twenty-four hours later, CHO cells were sub-cultured into a 96-well plate at 37°C for an additional 24 h before treatment. After removal of the medium from the 96-well plate, the cells were treated with 100 μl ligand-containing medium (or ligand-free medium) for 6 h. Finally, CHO cells were lysed with 1 × passive lysis buffer for luciferase assay (Promega) and the luciferase activity of the cell lysate was measured by a Multimode Microplate Reader (TriStar LB941, EG&G Berthold, Germany) according to the manufacturer’s instruction.

To test whether cMRAP1 and cMRAP2 can alter the pharmacological properties of cMC5R, CHO cells co-expressing MC5R and cMRAP1/cMRAP2 were treated with chicken ACTH_1–39_/α-MSH, and the relative potential of these two peptides to activate the receptor was also determined using the pGL3-CRE-luciferase reporter system. At the same time, H89 (a PKA inhibitor, 10 μM) and MDL12330 A (an AC inhibitor, 5 μM) were used to further determine the signal pathways activated by ACTH_1-39_/α-MSH.

### Western blot

To investigate whether the activation of cMC5R can enhance CREB phosphorylation, 100 ng of cMC5R expression plasmid, or an empty pcDNA3.1 (+) vector, was transfected into CHO cells cultured in a 24-well plate (Nunc) using jetPRIME transfection reagent (Polyplus Transfection). After 24 h transfection, ACTH (10 nM) or α-MSH (10 nM) was added to treat the cells for 10 min. Then the whole-cell lysates were used to examine the level of phosphorylated CREB using western blot. The level of total CREB protein was also examined and used as internal controls in each experiment. The phosphorylated CREB (p-CREB) and total CREB levels were quantified by densitometric analysis with ImageJ software. Relative optical densities of p-CREB normalized by CREB levels compared to the densities in the absence of ACTH (or α-MSH) are shown as relative p-CREB/CREB expression (fold changes).

### Detection of cell surface expression of MC5R by Nano-Glo HiBiT detection system

To quantify the cell surface expression of cMC5R, the Nano-Glo^®^ HiBiT Extracellular and Lytic Detection System purchased from Promega Corporation (Promega) were used according to our previously established methods (Zhang et al., 2020). Nano-Glo^®^ HiBiT Extracellular Detection System can quantify HiBiT-tagged MC5R expressed on the cell membrane, while the Lytic Detection System can determine the total HiBiT-tagged MC5R levels in cultured cells.

In brief, CHO cells cultured in a 96-well plate were transfected with HiBiT-tagged receptor (HiBiT-MC5R) expression plasmid (or co-transfected with HiBiT-MC5R and MRAP1 (or MRAP2) plasmids) and incubated for an additional 24 h. To quantify the cell surface expression of HiBiT-MC5R, 40 μl Nano-Glo^®^ HiBiT Extracellular Reagent, in which LgBiT protein can bind to HiBiT-MC5R expressed on the cell surface and generate luminescence, was added, and the luminescence values were measured by a Multimode Microplate Reader (TriStar LB 941, EG&G Berthold, Germany). To quantify the total expression levels of HiBiT-MC5R in cells, Nano-Glo^®^ HiBiT Lytic Reagent was added to lyse cells, and the luminescence values were also measured according to the manufacturer’s instruction. Finally, the relative cell-surface expression level of MC5R (HiBiT-MC5R signal on the cell membrane) was first normalized by the total HiBiT-MC5R signals in cells, and then expressed as the percentage to the control group.

### Tissue expression of chicken *MC5R*, *MRAP1*, and *MRAP2*

To examine the mRNA abundance of chicken *MC5R*, *MRAP1* and *MRAP2* in different domestic chicken tissues, we used a large-scale RNA-Seq dataset representing all the major organ systems from adult Lohmann White domestic chickens (Zhang et al., 2022). An open-access chicken tissue gene expression atlas (TGEA) (https://chickenatlas.avianscu.com/) is presented based on the expression of 224 samples across 38 well-defined chicken tissues. It allows us to view and download the expression profile of chicken *MC5R*, *MRAP1* and *MRAP2* across tissues. Expression levels were estimated in transcript per million (TPM) units. The raw data that support the findings of this RNA-Seq dataset have been deposited into CNGB Sequence Archive (CNSA) of China National GeneBank DataBase (CNGBdb) with accession number CNP0003404.

To examine the expression levels of chicken *MC5R* and *MRAP1* mRNA at different development stages, RNA-Seq data were downloaded from the SRA database (Accessions: PRJEB26695, University of Heidelberg). This dataset covers the development of liver organs from day 10 post-conception to day 155 post-hatch. The quantification of reads was performed with Salmon v1.4.0 (Patro et al., 2017) against the NCBI GRCg7b database (https://www.ncbi.nlm.nih.gov/assembly/GCF_016699485.2/). The transcripts per million (TPM) values were used to estimate the abundance of *MC5R* and *MRAP1* mRNA transcripts.

### Identification of the promoter regions of cMC5R

The promoter activity of these constructs was detected in DF1 cells, which is a continuous cell line of chicken embryo fibroblasts, by the dual-luciferase reporter assay (Promega, Madison, WI), as described in our previous study (He et al., 2016; Gao et al., 2017). In brief, DF-1 cells were cultured in a 48-well plate at a density of 1 × 10^5^ cells per well before transfection. After 24 h incubation, a mixture containing 100 ng of promoter construct, 5 ng of pRL-TK construct and 0.5 μl of JetPRIME (Polyplus-transfection, France) was prepared in 200 μl of buffer and transfected following the manufacturer’s instructions. 24 h later, the medium was removed and 100 μl 1 × passive lysis buffer (Promega) was added to each well. Luciferase activities of 15 μl of cellular lysates were measured using DLR assay kit (Promega). Luciferase activity of promoter-luciferase construct in DF-1 cells was normalized to *Renilla* luciferase activity derived from the pRL-TK vector (Promega). Then, the luciferase activities in each treatment group were expressed as fold change as compared with the control group. The cells transfected with the empty pGL3-Basic vector was used as an internal control group.

### Evaluation of the effect of ACTH_1-39_ on primary cultured chicken hepatocytes

To evaluate the effect of ACTH_1-39_ on chicken liver, the chicken hepatocytes were prepared and maintained as a monolayer culture. Briefly, one-month-old male chicken hepatocytes were isolated by perfusion of a liver with Ca^2+^-free KRB buffer to remove blood cells, and being digested with 0.25% collagenase-I for 10 min. Hepatocytes were obtained following filtering and washing using M199 medium. Then the hepatocytes were plated in a Corning CellBIND 48-well microplate (Corning, NY, cat. no.3338) with M199 medium containing 100 U/ml penicillin and 100 mg/ml streptomycin (Gibco), 5 μg/ml bovine insulin (Sigma), and 5% FBS at 37°C in a 5% CO_2_ atmosphere. To adhere to the wall, the cells were incubated on the 48-well plate for 4 h.

Glucose production from primary cultured chicken hepatocytes was measured as previously described (Collins et al., 2006). Briefly, cells were washed three times with warm phosphate-buffered saline (PBS) to remove glucose, followed by treatment with 100 nM (or different concentration gradients) chicken ATCH_1-39_ for 30 min or 60 min in glucose-free medium containing gluconeogenic substrates (20 mM sodium lactate and 2 mM sodium pyruvate). Glucose concentration was determined with a glucose assay kit (Applygen, Beijing, China, catalog no. E1011). Briefly, the 5 µl medium was added into 195 µl reagent and incubated for 20 min at 37°C. Absorbance in 550 nm was measured in a standard glucose solution.

For lipid quantification by Oil Red O staining, after treatment with ACTH_1-39_ for 24 h, primary cultured chicken hepatocytes were washed with PBS and then fixed with 4% paraformaldehyde for 30 min. After PBS washing, the Oil Red O working solution was added to stain for 30 min. The stained lipids were then visualized by light microscopy after washing in PBS. To quantify the lipid content, the Oil Red O stained in the cells was extracted with isopropanol by measuring the OD value at 540 nm.

For measurement of triglyceride content, after treated with ACTH_1-39_ for 24 h, hepatocytes were harvested and triglyceride (TG) content was analyzed using a commercial triglyceride content assay kit (Applygen, Beijing, China, catalog no. E1013). Results were normalized to the protein content of each sample, as determined using a BCA assay kit (Beyotime Institute of Biotechnology, China, catalog no. P0010).

For quantitative real-time PCR, primary cultured chicken hepatocytes were treated with various concentrations of ACTH_1-39_ (or other composition) at 37°C for 6 h. The relative amount of mRNA was calculated using the comparative Ct method. Chicken β-actin gene was used as the reference gene. Amplification of specific transcripts was confirmed by analyzing the melting curve profile performed at the end of each run and by determining the size of the PCR products using agarose electrophoresis and ethidium bromide staining.

### Data analysis

All statistical analysis was performed using GraphPad Prism 9 (Graph Pad Software Inc., San Diego, CA). The dose-response curves were constructed using nonlinear regression models. Student’s test was used to compare two groups, for more than two groups one-way ANOVA was performed followed by Dunnett’s test. To validate our results, all *in-vitro* experiments were repeated three times, and representative data are reported.

## Results

### Characterization of the chicken *MC5R* full-length cDNA

**FIGURE 1:**
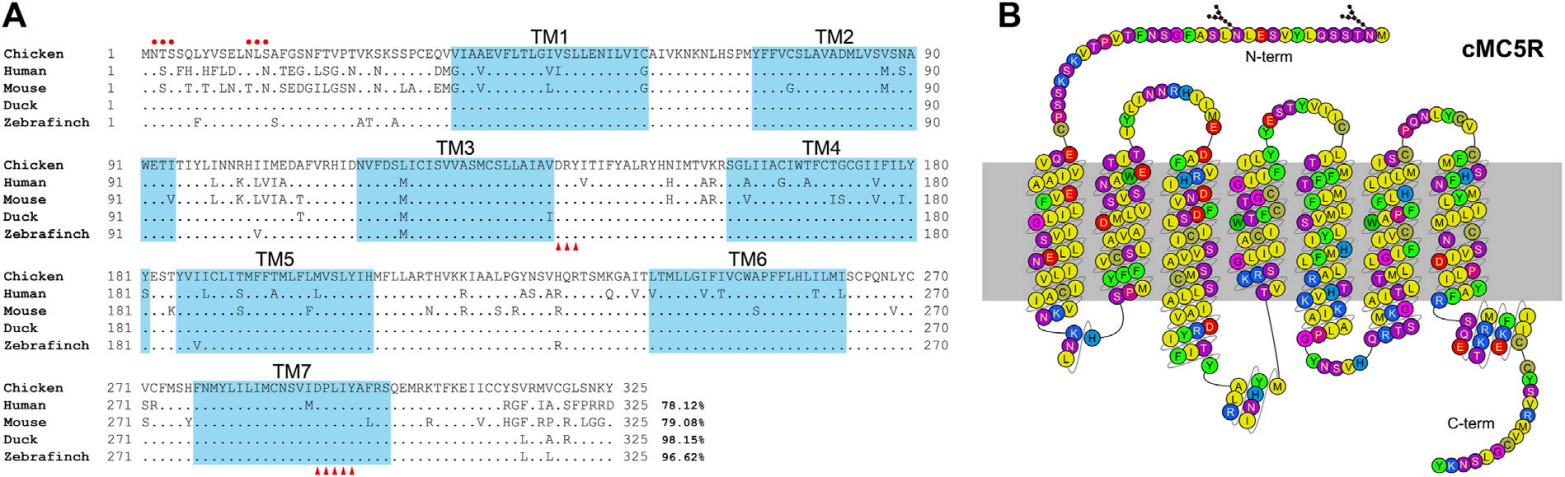
**(A)** Alignment of mature MC5R amino acid sequences among five species. NCBI accession numbers of these sequences are OP259502 (chicken), NP_005904.1 (human), NP_038624.3 (mouse), XP_005018248.1 (duck) and XP_004175266.1 (zebrafinch). The TM1-7 (seven transmembrane domains) are shaded in blue. The red dots indicate the predicted N-glycosylation sites. The conserved DRY motif and DPXXY motif were marked with red triangles. “.” represents identical amino acid, and “−” indicates a gap in the sequence alignment. **(B)** Snake-like diagram of the chicken MC5R receptor. Residues are colored based on their chemical properties. N-term represents the N terminal sequence, C-term represents the C terminal sequence.

According to the predicted cDNA sequences of *MC5R* deposited in GenBank (KF670718.1), we amplified and cloned the cDNAs of *MC5R* from chicken liver tissue. The chicken full-length *MC5R* gene contains two exons (GenBank accession no. OP259502), and its 5′-untranslated region (5′-UTR) and 3′-UTR is 253 bp and 601 bp in length, respectively. It is predicted to encode a G protein-coupled receptor of 325 amino acids (Figure 1).

Amino acid sequence alignment of chicken MC5R with their corresponding orthologs in other vertebrate species was shown in Figure 1A. According to multiple sequence alignment, the deduced amino acid sequence of MC5R is similar to the sequences of other known MC5Rs with a high degree of amino acid sequence identity to that of human (78%), mouse (79%), duck (98%) and zebrafinch (97%). The C-terminal and the seven transmembrane domains (TM1-TM7) of these MC5R sequences are more similar than the N-terminal. Further analysis of MC5R sequences revealed several structural features, including two putative N-glycosylation sites (NXS/T, where X stands for any amino acid except proline), an Asp-Arg-Tyr (DRY) motif at the bottom of TM3, and a highly conserved Asp-Pro-X-X-Tyr (DPXXY) motif at the TM7 (Figure 1B).

### Functional characterization of chicken MC5R

**FIGURE 2:**
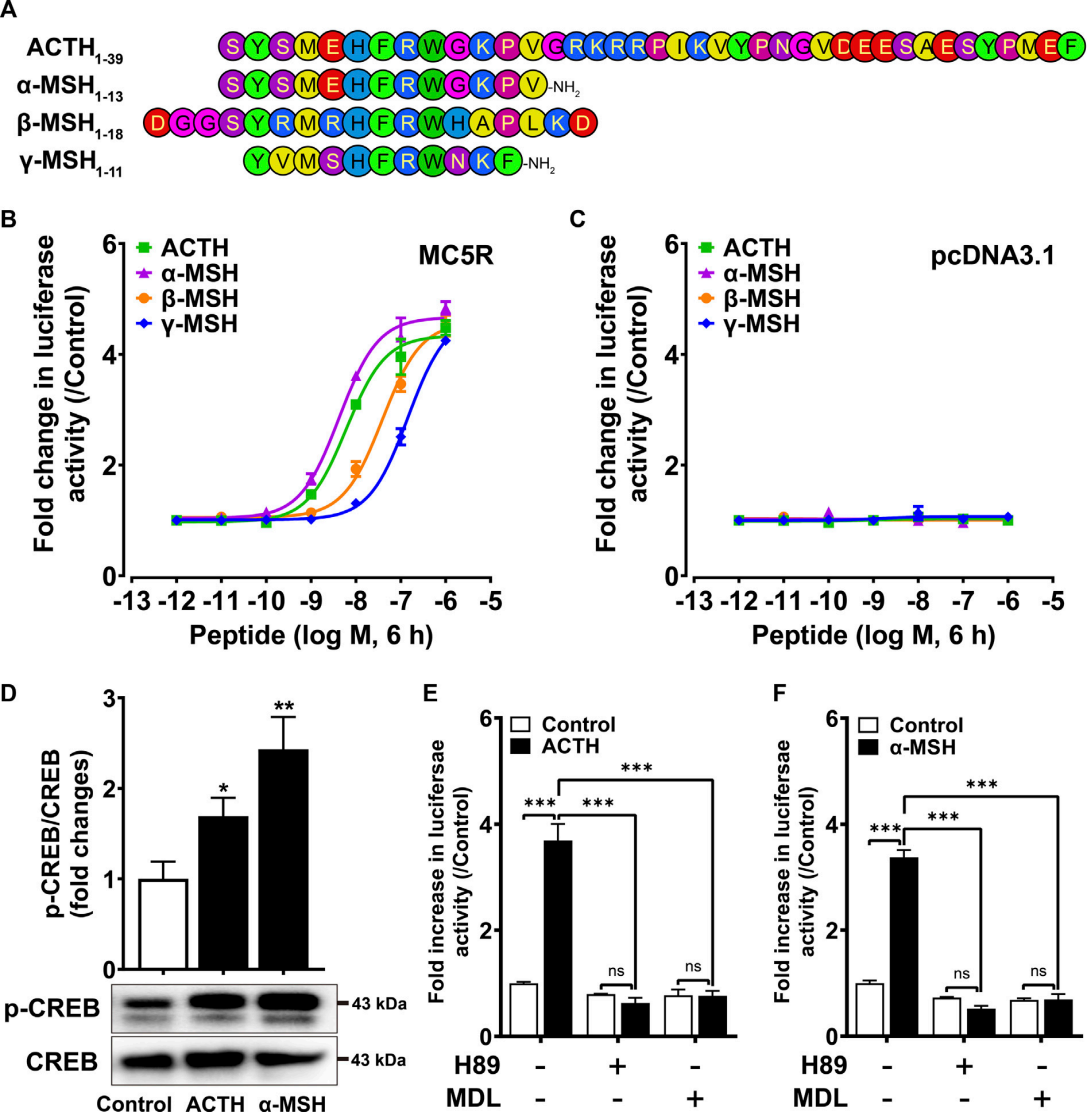
**(A)** Amino acid sequences of chicken ACTH, a-MSH (acetyl-a-MSH with an amidated C-terminus), β-MSH and γ-MSH (acetyl-γ-MSH with an amidated C-terminus) used in this study. **(B)** Effects of ACTH, a-MSH, β-MSH and γ-MSH in activating chicken MC5R expressed in Chinese hamster ovary (CHO) cells, as monitored by the pGL3-CRE-luciferase reporter system. Data are shown as the mean ± SEM of three replicates (*N* = 3) and are representative of three independent experiments. **(C)** Effects of ACTH, a-MSH, β-MSH and γ-MSH in activating negative control pcDNA3.1 expressed in CHO cells. Data are shown as the mean ± SEM of three replicates (*N* = 3) and are representative of three independent experiments. **(D)** Both ACTH and a-MSH treatment (10 nM, 10 min) could enhance CREB phosphorylation levels of CHO cells expressing cMC5R. The phosphorylated CREB (p-CREB) levels were quantified by densitometric analysis, normalized by that of cellular total CREB, and expressed as fold difference compared to the control (0 min). Data points represent the mean ± SEM of three independent experiments performed in triplicate. The representative set of Western blots is shown at the bottom. *, *p* < 0.05, **, *p* < 0.01 vs. control. **(E–F)** Effects of H89 (10 μM) and MDL (5 μM) on ACTH (10 nM, 6 h) **(E)** or a-MSH (10 nM, 6 h) **(F)** induced luciferase activities of CHO cells expressing cMC5R, monitored by pGL3-CRE-luciferase reporter system. H89 or MDL was added 0.5 h before treatment. Each figure shows one representative experiment repeated three times. ***, *p <* 0.001, ns, non-significant.

To investigate the functionality of chicken MC5R under stimulation with synthetic chicken melanocortin peptides (α-, β-, γ-MSH and ACTH_1-39_) (Figure 2A), the pGL3-CRE-luciferease reporter system was used to monitor receptor-stimulated intracellular cAMP/PKA signaling pathway (Zhang et al., 2017). All four chicken melanocortin peptides were shown to potently activate cMC5R in CHO cells (Figure 2B). Among the four ligands, ACTH and α-MSH had the highest potency to activate cMC5R, with similar EC50 values (6.12 ± 1.18 nM and 4.23 ± 0.83 nM). The EC50 values for β-MSH and γ-MSH upon activation of cMC5R were 38.7 ± 6.51 nM and 144.4 ± 15.41 nM, respectively, which were approximately 10-fold and 30-fold lower than ACTH/α-MSH. In addition, the control plasmid pcDNA3.1 expressed in CHO cells did not respond to treatment with chicken melanocortin peptides (Figure 2C).

We also investigated the effect of cMC5R activation on CREB phosphorylation. Western blotting was used to show that CREB phosphorylation (43 kDa) was significantly increased when CHO cells expressing cMC5R were stimulated with 10 nM ACTH and 10 nM α-MSH for 10 min (Figure 2D). To further confirm the functional coupling of cMC5R to the intracellular cAMP/PKA signaling pathway, inhibitors targeting the intracellular cAMP/PKA signaling pathway were used to test whether they might inhibit the receptor-activated signaling pathway. H89 (a PKA inhibitor, 10 μM) and MDL12330 A (an AC inhibitor, 5 μM) could significantly inhibit ACTH-stimulated (Figure 2E) and α-MSH-stimulated (Figure 2F) luciferase activity in CHO cells expressing cMC5R, confirming the functional coupling of cMC5R to the AC/cAMP/PKA signaling pathways.

### Interaction of chicken MRAP1 and MRAP2 with MC5R

**FIGURE 3:**
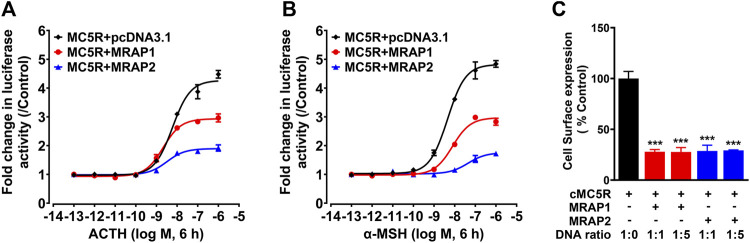
**(A)** Effect of ACTH in activating chicken MC5R expressed in CHO cells co-transfected with cMRAP1 or cMRAP2 expression plasmid, as monitored by the pGL3-CRE-luciferase reporter system. **(B)** Effect of a-MSH in activating chicken MC5R expressed in CHO cells co-transfected with cMRAP1 or cMRAP2 expression plasmid, as monitored by the pGL3-CRE-luciferase reporter system. Data are shown as the mean ± SEM of four replicates (*N* = 4) and are representative of three independent experiments. **(C)** Surface expressions of chicken MC5R in CHO cells transfected with MC5R and MRAP1 (or MRAP2) at the indicated ratio were measured by the HiBiT-tagging extracellular detection system (Promega) and the total expression levels of HiBiT-MC5R measured by HiBiT lytic assay were used as an internal control to normalize transfection efficiencies. Each figure shows one representative experiment repeated three times. ***, *p <* 0.001.

To investigate the influences of chicken MRAPs on the responsiveness of cMC5R to α-MSH and ACTH, CHO cells co-expressing cMC5R and cMRAPs were treated with ACTH_1–39_ and α-MSH, and the receptor activation was monitored by pGL3-CRE luciferase reporter system. Following stimulation with chicken ACTH_1-39_, the EC50 value for MC5R expressed alone was 5.91 ± 1.18 nM, whereas the EC50 values for MC5R co-expressed with cMRAP1 or cMRAP2 were 2.12 ± 0.37 nM and 3.19 ± 1.09 nM, respectively (Figure 3A). It indicated that either MRAP1 or MRAP2 had no effect on the sensitivity of chicken MC5R for ACTH. Following stimulation with α-MSH, the EC50 value for MC5R expressed alone was 4.23 ± 0.83 nM, whereas the EC50 values for MC5R co-expressed with cMRAP1 or cMRAP2 were 7.85 ± 1.58 nM or 44.2 ± 10.21 nM, respectively (Figure 3B). Notably, co-expression of MRAP2 significantly reduced the sensitivity of MC5R to α-MSH (Table 1).

**TABLE 1:** EC_50_ values of chicken ACTH_1-39_ and α-MSH in activating cAMP/PKA signaling pathways in CHO cells expressing chicken MC5R.

EC_50_ (nM)	MC5R	MC5R + MRAP1	MC5R + MRAP2
ACTH_1-39_	5.91 ± 1.18	2.12 ± 0.37	3.19 ± 1.09
α-MSH	4.23 ± 0.83	7.85 ± 1.58	44.20 ± 10.21^a^

Results were expressed as the mean ± SEM, of at least three independent experiments.

a Significantly different from the parameter of MC5R, *p* < 0.01.

To test whether MRAP1 and MRAP2 could alter the trafficking of MC5R, we measured the surface expression of chicken MC5R in the absence or presence of cMRAPs using Nano-Glo HiBiT Detection System (Figure 3C). Compared with CHO cells transfected with MC5R only (1:0), the expression of chicken MC5R on the cell surface was significantly reduced to about 25% in the presence of MRAP1 or MRAP2 at a progressive ratio (1:1 and 1:5). This finding clearly indicated that MRAP1 and MRAP2 inhibit the cell surface expression of chicken MC5R.

### Tissue expression of chicken *MC5R*, *MRAP1*, and *MRAP2*

**FIGURE 4:**
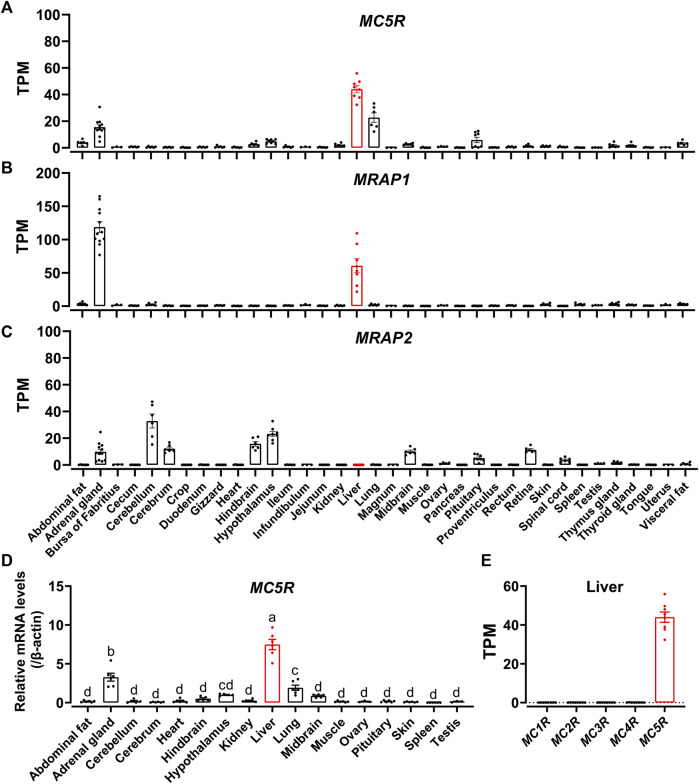
**(A–C)** RNA-seq data analysis showed the expression of *MC5R* **(A)**, *MRAP1* **(B)**, and *MRAP2* **(C)** in adult Lohmann Layer strain chicken tissues. Each dot represents an individual. The transcripts per million (TPM) values were used to estimate the abundance of mRNA transcripts. **(D)** Quantitative real-time PCR assay of *MC5R* mRNA levels in Lohmann Layer strain chicken tissues, including the cerebellum, midbrain, cerebrum, hindbrain, hypothalamus, anterior pituitary, kidney, liver, lung, muscle, skin, testis, abdominal fat, heart, spleen, and ovary. The mRNA levels of target genes were normalized to that of β-actin and expressed as the fold difference compared with that of the midbrain. Each data point represents the mean ± SEM of six adult chickens (*N* = 6, three males and three females, one-year-old), except for that of ovary and testis, which represent the mean ± SEM of three adult chickens (*N* = 3). Different superscripts **(A–D)** among the different developmental stages are significantly different (*p* < 0.05) by one-way ANOVA test, followed by Dunn’s multiple comparison test. **(E)** The mRNA expression of five MCRs in the Lohmann Layer liver tissue using transcriptome data from our gene expression atlas (https://chickenatlas.avianscu.com/). The transcripts per million (TPM) values were used to estimate the abundance of mRNA transcripts.

To examine the tissue distribution of *MC5R*, *MRAP1* and *MRAP2* in adult chickens, we analyzed the expression in 36 chicken tissues with reference to the RNA-seq data previously obtained in our lab. We found that *MC5R* was widely expressed in various tissues, including liver, lung, adrenal gland, anterior pituitary, abdominal fat, visceral fat, hypothalamus, and kidney (Figure 4A). The highest level of *MC5R* transcript was detected in the liver. *MRAP1* was abundantly expressed in the adrenal gland and liver as illustrated in Figure 4B. RNA-seq results showed that *MRAP2* was also highly expressed in the cerebellum, hypothalamus, hindbrain, cerebrum, midbrain, adrenal gland, retina, and anterior pituitary, weakly expressed in the thymus gland, spinal cord, testis, and fat, and with almost undetectable expression in the liver (Figure 4C).

Using quantitative reverse transcription PCR (RT-qPCR), we re-examined the mRNA expression of *MC5R* in adult chicken tissues, including the cerebellum, midbrain, cerebrum, hindbrain, hypothalamus, anterior pituitary, kidney, liver, lung, muscle, skin, testis, adrenal gland, abdominal fat, heart, spleen, and ovary. In agreement with the RNA-Seq data, *MC5R* transcript had the significantly highest abundance in the liver, which was about 2.3-fold and 3.5-fold higher than those detected in the adrenal gland and lung (Figure 4D). Transcripts of *MC5R* have also been detected weakly in other tissues, including anterior pituitary, skin, muscle, kidney, testis, fat, ovary, and some brain regions. In addition, the expression of the other four MCRs were also examined *via* the RNA-Seq data atlas (Figure 4E), and only *MC5R* could be detected in adult chicken liver tissue, while the TPM values of the other MCRs were almost zero.

**FIGURE 5:**
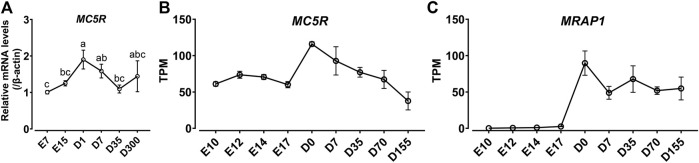
**(A)** Quantitative real-time PCR assay of *MC5R* mRNA levels in Lohmann Layer strain chicken tissues at different development stages, including embryonic day 7 (E7), embryonic day 15 (E15), day 1 after hatch (D1), day 7 after hatch (D7), day 35 after hatch (D35) and day 300 after hatch chicken (D300). The mRNA levels of target genes were normalized to that of β-actin and expressed as the fold difference compared with that of embryonic day 7 (E7). Each data point represents the mean ± SEM of six adult chickens (*N* = 6, three males and three females). Different superscripts (a–c) among the different developmental stages are significantly different (*p* < 0.05) by one-way ANOVA test, followed by Dunn’s multiple comparison test. **(B–C)** The mRNA expression of *MC5R* **(B)** and *MRAP1* **(C)** genes in the red junglefowl (*Gallus gallus*) at different developmental periods using transcriptome data from the NCBI public database (Accessions: PRJEB26695). This dataset covers the development of liver organ from day 10 post-conception to day 155 post-hatch. The transcripts per million (TPM) values were used to estimate the abundance of mRNA transcripts.

Since *MC5R* is highly expressed in the liver, we examined the mRNA expression of *MC5R* in liver at different developmental stages, including at embryonic day 7 (E7), embryonic day 15 (E15), day 1 after hatch (D1), day 7 after hatch (D7), day 35 after hatch (D35) and day 300 after hatch chicken (D300). As shown in Figure 5A, expression of *MC5R* gradually increased from day 7 of embryonic development and reached its peak significantly on day 1 after hatch. During the post-hatch growth period, *MC5R* expression was downregulated significantly.

In addition, we analyzed the expression trends of *MC5R* and *MRAP1* genes in the red junglefowl (*Gallus gallus*) at different developmental periods using transcriptome data from the NCBI public database (Accessions: PRJEB26695). In high agreement with the quantitative PCR results in Lohmann Layer strain, *MC5R* was expressed at the highest level in chicken liver at day 0 after hatch, and *MC5R* expression gradually decreased after hatch (Figure 5B). Unexpectedly, *MRAP1* expression was barely detectable in chicken embryos, while *MRAP1* had the highest expression level at day 0 after hatch and then maintained a stable expression level in the chicken liver (Figure 5C).

### Analysis of the chicken *MC5R* promoter region

To identify the promoter region of chicken *MC5R*, we constructed several promoter-luciferase constructs containing the 5′ flanking region of *MC5R* with different lengths and tested their promoter activities in cultured DF1 chicken embryonic fibroblasts. A 1998 bp PCR fragment containing 171 bp of *MC5R* cDNA sequence (exon 1) and 1827 bp of 5′-flanking sequence was first obtained by genomic PCR. To determine the core promoter region of *MC5R*, four promoter-luciferase reporter constructs of chicken *MC5R* were generated by cloning the 1.9 kb fragment or its truncated fragment into pGL3-basic vector.

**FIGURE 6:**
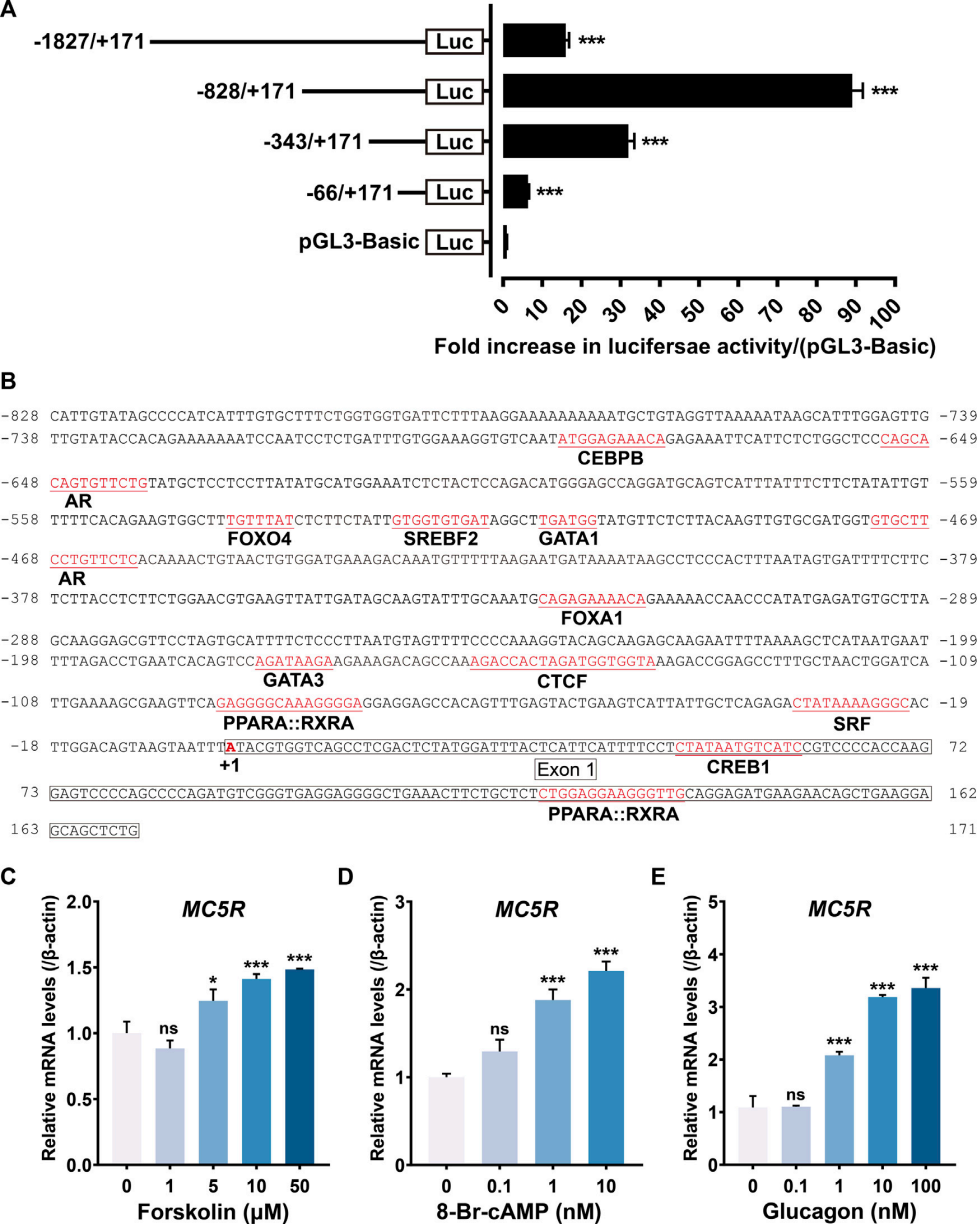
**(A)** Detection of the promoter activities of the 5′-flanking regions of chicken *MC5R* gene in cultured DF-1 cells. Various stretches of the 5′-flanking regions of chicken *MC5R* were cloned into a pGL3-Basic vector for the generation of four promoter-luciferase constructs (P1: −1827/+171-Luc, P2: −828/+171-Luc, P3: −343/+171-Luc, P4: −66/+171-Luc). Their promoter activities were determined by the Dual-Luciferase Reporter (DLR) assay. All experiments were performed in triplicate and represent at least three independent biological repeats. Data shown represent mean ± SEM. ***, *p* < 0.001 vs. promoter-less pGL3-Basic vector. **(B)** Partial sequence (-828/+171) of the chicken *MC5R* promoter region. The predicted binding sites for transcriptional factors, such as CREB1, SRF, CTCF, AR, FOXA3, FOXA1, and CEBPB were shaded. The transcriptional start site ‘A’ was marked with red and designated as ‘+1’. The sequences of exon 1 was boxed. **(C–E)** Effects of forskolin **(C)**, 8-Br-cAMP **(D)** and chicken glucagon **(E)** with different concentrations on *MC5R* gene expression in cultured chicken primary hepatocytes examined by RT-qPCR. Each data point represents means ± SEM of four replicates (*N* = 4). *, *p* < 0.05, ***, *p* < 0.001, ns, non-significant vs. respective control (without peptide treatment).

As shown in Figure 6A, the 5′-flanking regions of *MC5R* from -1827 to +171 (P1) exhibited promoter activity in DF1 cells. When the 5′-end of P1 was truncated (P2: −828/+171), the activity of luciferase in DF1 cells was greatly increased. Interestingly, continued truncation of the 5′-end fragments of P2 (P3: −343/+171 and P4: −66/+171) reduced the promoter activity. We also noted that although the length of P3 was 514 bp, it had a 32-fold increase in luciferase activity compared to the control vector, indicating that this region (-343 to +171) retains the essential minimum activity. Using the online software AnimalTFDB (v3.0) (Hu et al., 2019), the putative binding sites for many transcription factors, including CREB1, SRF, CTCF, AR, FOXA3, FOXA1, PPARX:RXRA and CEBPB were predicted to exist within or near the promoter region P2 (Figure 6B).

To clarify whether the cAMP/PKA/CREB pathway stimulates *MC5R* transcription or not, we examined the effect of forskolin (an adenylyl cyclase activator), 8-Br-cAMP (a cell-permeable cAMP analog, PKA activator) and glucagon (induce cAMP elevation in a dose-dependent way) on the expression level of *MC5R* in primary chicken hepatocytes. Treatment of hepatocytes with forskolin significantly increased *MC5R* mRNA abundance (Figure 6C). Incubating hepatocytes with 8-Br-cAMP also stimulated a dose-dependent increase in *MC5R* mRNA abundance (Figure 6D). Incubating chicken hepatocyte cultures with glucagon (10–100 nM) stimulated a 3.5-fold increase in *MC5R* mRNA abundance after 6 h of treatment (Figure 6E). These results demonstrated that glucagon increased hepatic *MC5R* mRNA abundance and provided evidence that the PKA branch of the cAMP pathway may play a role in mediating this effect.

### Effect of ACTH on glucolipid metabolism in primary hepatocytes

**FIGURE 7:**
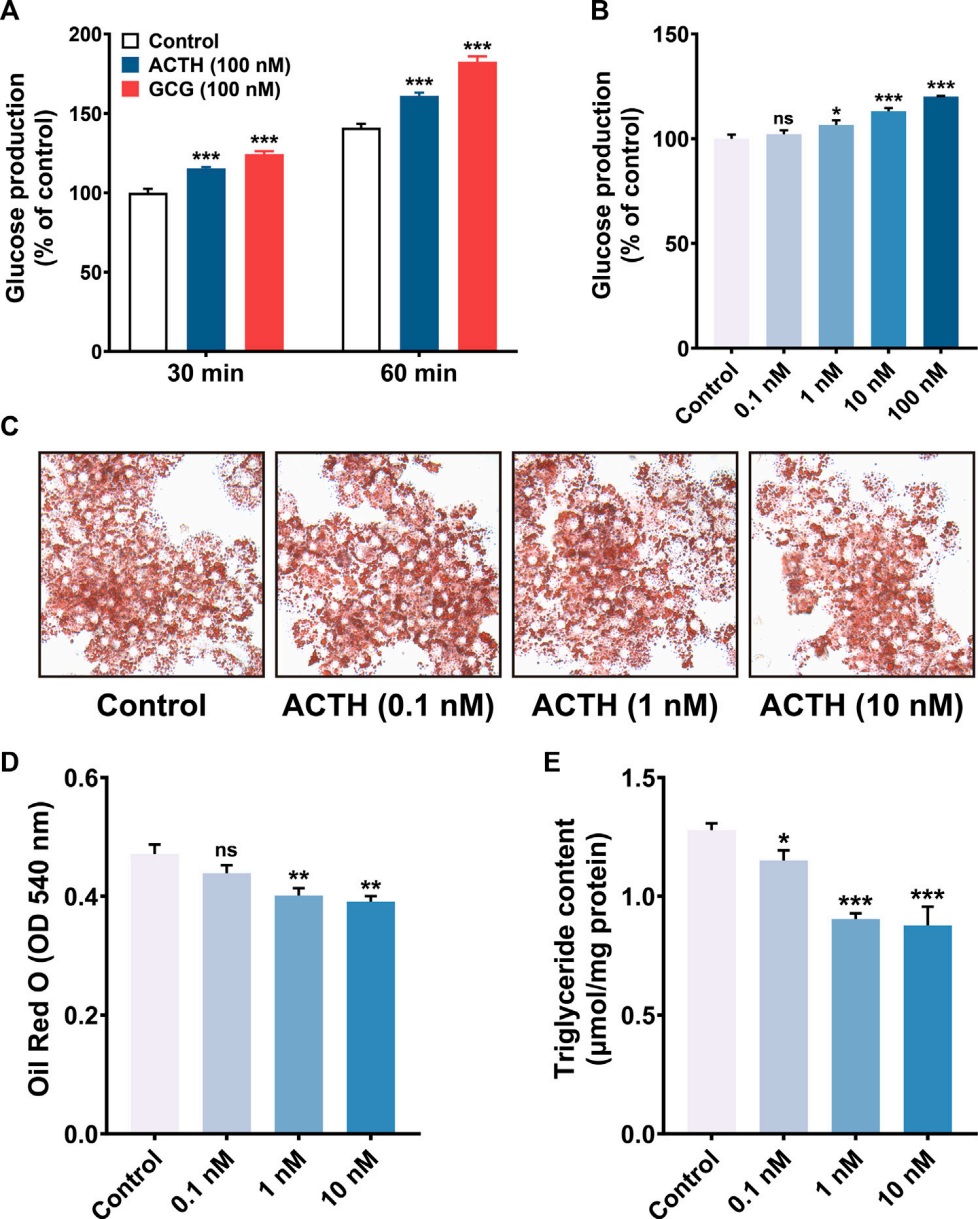
**(A)** ACTH and glucagon (GCG) induction of gluconeogenesis in primary cultured chicken hepatocytes. Primary cultured chicken hepatocytes were incubated with 100 nM of ACTH and GCG for various times (30 and 60 min), glucose production was then measured. Values represent means ± SEM of four independent experiments with triplicate dishes. ***, *p* < 0.001 vs. control. **(B)** ACTH induction of gluconeogenesis in primary cultured chicken hepatocytes. Primary cultured chicken hepatocytes were incubated with different concentrations (0.1–100 nM) of ACTH for 60 min. Values represent means ± SEM of four independent experiments with triplicate dishes. *, *p* < 0.05, ***, *p* < 0.001, ns, non-significant. **(C–D)** Oil red O staining (magnification: 10×40) and quantification analysis (*N* = 6), red circle drops mean fat droplets. The Oil Red O stained in the cells was extracted with isopropanol by measuring the OD value at 540 nm **, *p* < 0.01, ns, non-significant vs. control. **(E)** The effects of ACTH on triglyceride content in primary chicken hepatocytes. *, *p* < 0.05, ***, *p* < 0.001 vs. control.

Both *MC5R* and *MRAP1* were highly expressed in chicken liver after hatch, which indicated that the liver might be another target organ for ACTH, in addition to the adrenal gland, when the chicken HPA axis is active. We then evaluated the influence of cACTH_1-39_ on the glucose production and lipid contents of primary hepatocytes to explore the biological roles of ACTH mediated by MC5R and MRAP1 in the chicken liver. As shown in Figures 7A,B, cACTH_1-39_ significantly increased the level of glucose production in a dose (≥1 nM)- and time (≥0.5 h)-dependent manner in primary hepatocytes. When compared with the control, ACTH prominently decreased triglyceride (TG) contents, oil red O staining showed that lipid contents were lower in the ACTH-treat group in a dose-dependent manner (Figures 7C–E).

**FIGURE 8:**
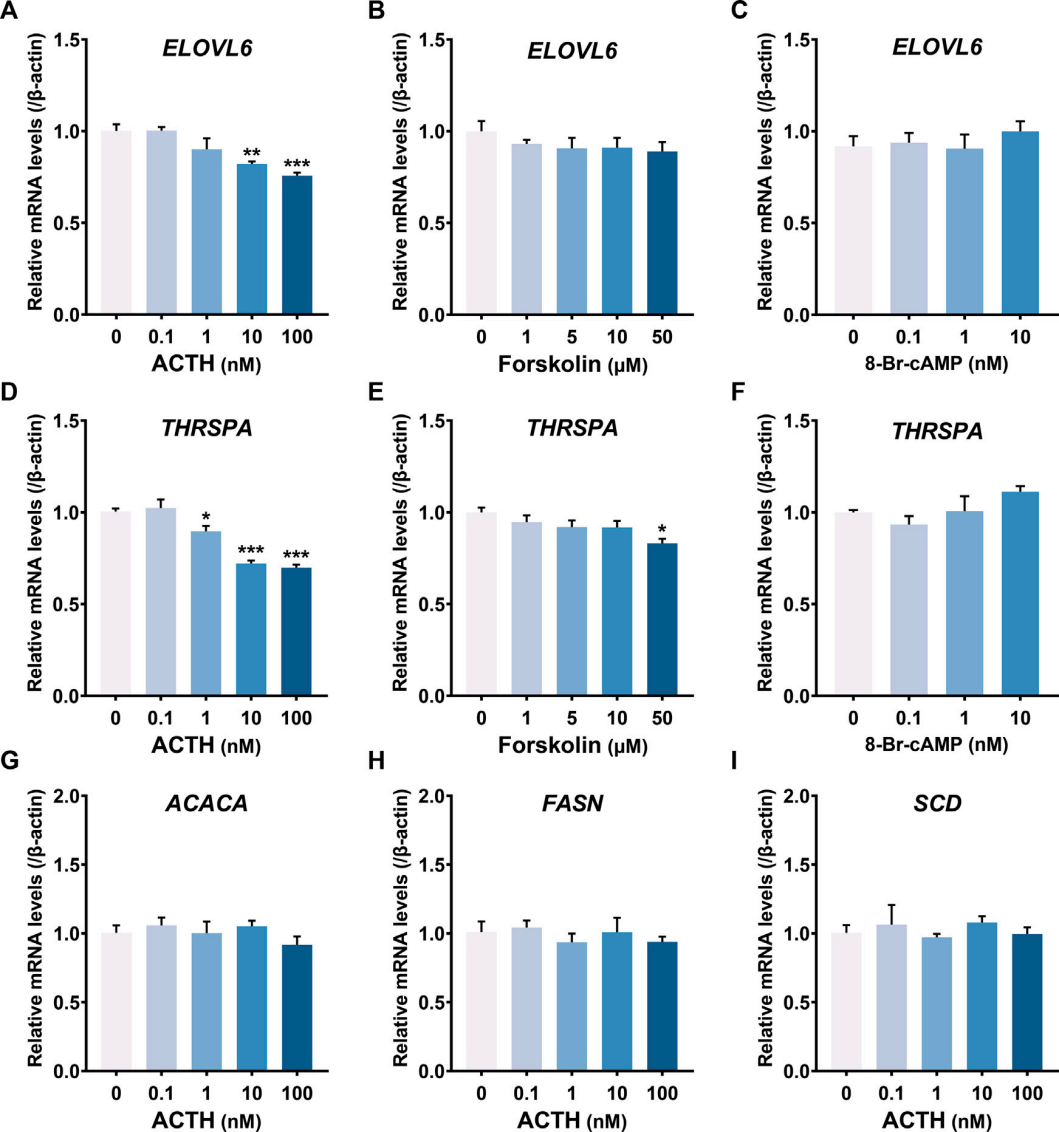
**(A–F)** Effects of chicken ACTH_1-39_, forskolin and 8-Br-cAMP with different concentrations on gene expression, including *ELOVL6* **(A–C)** and *THRSPA* **(D–F)**, in cultured chicken primary hepatocytes examined by RT-qPCR. **(G–I)** Effects of chicken ACTH_1-39_ with different concentrations (0.1–100 nM) on gene expression, including *ACACA* **(G)**, *FASN* **(H)** and *SCD* **(I)**, in cultured chicken primary hepatocytes. Each data point represents means ± SEM of four replicates (*N* = 4). *p < 0.05, **p < 0.01, ***p < 0.001 vs. respective control (without peptide treatment)..

In addition, ACTH dose-dependently decreased *ELOVL6* (Figure 8A) and *THRSPA* (Figure 8D) mRNA expression, instead of *ACACA*, *FASN* and *SCD* (Figures 8G–I), which were all related to lipogenesis. The expression level of *ELOVL6* and *THRSPA* in primary hepatocytes was not affected by Forskolin (adenylyl cyclase activator) (Figures 8B,E) and 8-Br-cAMP (PKA activator) in a dose-dependent way (Figures 8C,F). These results demonstrated that ACTH may relieve triglyceride deposition by inhibiting *de novo* lipogenesis.

## Discussion

In this study, *MC5R* cDNAs were cloned from the chicken liver. The current work provided evidence for the promoter activity of MC5R in chickens and confirmed that MC5R was sensitive to multiple endogenous melanocortin ligands in chickens. The effects of chicken MRAP1 and MRAP2 on MC5R signaling and trafficking were also discussed. Based on transcriptomic and qPCR data, MC5R was highly expressed in the liver along with MRAP1 in post-hatch chickens. Then we found that chicken ACTH_1-39_ enhanced glucose production and decreased triglyceride contents in primary hepatocytes, dose-dependently. Gene expression studies revealed that *ELOVL6* and *THRSPA* were down-regulated, suggesting ACTH supplementation could suppress *de novo* lipogenesis. These results indicating that ACTH may play a direct role in hepatic metabolism. Previously, little information was available on the physiological functions of chicken MC5R, and our findings provided new evidence to explore the physiological roles of MC5R in avian.

Here, from the chicken brain, we cloned the full-length cDNA sequence of MC5R. Similar to cMC2R, the cMC5R transcript has an upstream non-coding exon and a coding exon with a long 3′-UTR. Both the MC2R and MC5R genes in chickens contain two exons, adding to the evidence that the MC2R and MC5R genes evolved from a common ancestor (Klovins et al., 2004; Dores, 2013). Sequence alignment showed that MC5Rs in different species all consisted of 325 amino acids with sequence similarity higher than 78% and were highly conserved between chicken and other birds or with human. The transmembrane regions of chicken MC5R were highly conserved to those of other vertebrates, but the N-terminal region differed considerably. This is consistent with earlier findings, which indicated that the MCR’s binding domain is likely to be present inside TM1, TM2, TM3, TM6, and TM7 (Schioth, 2001).

The luciferase reporter system was then employed to investigate the signal pathway of MC5R coupling. In response to α-MSH or ACTH, we demonstrated that the chicken MC5R can functionally couple to the Gs-linked cAMP/PKA signaling pathway, which is consistent with previous findings in human (Fathi et al., 1995), mice (Haskell-Luevano et al., 2001), blunt snout bream (Liao et al., 2019), zebrafish (Zhu et al., 2019), fugu (Klovins et al., 2004), sea bass (Sanchez et al., 2009), whale shark (Hoglin et al., 2020), and gar (Wolverton et al., 2019). Human peptides have previously been widely employed in the investigation of chicken MC5R (Ling et al., 2004; Thomas et al., 2018; Min et al., 2019; Dores et al., 2020). α-MSH is conserved in all positions between chicken and human, but the substitution of R15K and I20 V occurred in the KKRRPV motif of hACTH_1-24_. It was concluded that there were no statistical differences in the EC50 values when stimulated with either hACTH_1-24_ or srACTH_1–24_ (R15 and I20) (Dores et al., 2020). The synthesized chicken ACTH_1-39_, α-MSH (acetyl-α-MSH), β-MSH and γ-MSH were used in our study to confirm the pharmacological impact of ligands acting on the MC5R. Remarkably, our results showed that the ability of melanocortin to activate adenylate cyclase is ranked in the following order: ACTH_1-39_ ≈ α-MSH > β-MSH > γ-MSH. The ability of hACTH_1-39_ and its shortened form, hACTH_1-24_, to activate human and chicken MC5R has been shown to be no different in previous studies (Schioth et al., 1997; Ling et al., 2004). Our results were consistent with an early study that showed human ACTH_1-24_ and α-MSH had equal potency for chicken MC5R (Ling et al., 2004). The intracellular response of cMC5R to β-MSH and γ-MSH was first investigated in chicken. In contrast to ACTH_1-39_/α-MSH, the EC50 values of β-MSH and γ-MSH were approximately 10-fold and 30-fold lower, which is consistent with previous studies on human MC5R (Fathi et al., 1995) and blunt snout bream MC5R (Liao et al., 2019).

Melanocortin receptor accessory proteins (MRAP1 and MRAP2) were involved in the regulation of trafficking and signaling of vertebrate melanocortin receptors (Chan et al., 2009; Asai et al., 2013; Ramachandrappa et al., 2013; Zhang et al., 2017). As revealed in Figure 3, chicken MRAP1/MRAP2 did not affect the sensitivity of cMC5R to ACTH_1-39_, while MRAP2, instead of MRAP1, significantly decreased the sensitivity of cMC5R to α-MSH. At the same time, we observed that co-expression of MRAP1/MRAP2 with MC5R could reduce the plateau phase of the sigmoid curve, which is nicely correlated to the drop in membrane MC5R detected with MRAP1/MRAP2 co-expression. Using the Nano-Glo HiBit detection system, which is a novel method that allows quantitative and sensitive measurement of total and cell surface receptor expression (Rouault et al., 2017a; Reyes-Alcaraz et al., 2018), we further verified that both MRPA1 and MRAP2 could significantly decrease cell surface expression of MC5R compared to control, even at a 1:1 ratio.

Our results were consistent with previous results. In human, MRAPs were thought to down-regulate the cell surface expression of MC5R *in vitro*, and MRAP2 reduced the efficacy of hMC5R to NDP-MSH (Chan et al., 2009). It was reported that mouse MRAP1 had a negative effect on the surface expression of the MC5R receptor (Sebag and Hinkle, 2009). In zebrafish, MRAP2a decreased the surface expression and efficacy of MC5Ra/MC5Rb, but not MRAP2b, in the same manner as mouse MRAP2 did (Zhu et al., 2019). In ricefield eel (*Monopterus albus*), two isoforms of MRAP2 (maMRAP2X1 and maMRAP2X2) significantly decreased efficacy of maMC5R (Liu et al., 2021). In gar (*Lepisosteus oculatus*), MRAP2 and MRAP1 did not affect the sensitivity of MC5R, but increased the trafficking of MC5R to the plasma membrane (Wolverton et al., 2019).

However, our findings are in sharp contrast to the previous observations on chicken MC5R, which showed that chicken cMRAP1 significantly increased the sensitivity of cMC5R to hACTH_1–24_ (Thomas et al., 2018). Their further study confirmed this result and suggested that when cMC5R was co-expressed with cMRAP1, the KKRRP motif of hACTH_1–24_ is necessary to increase the sensitivity of MC5R to hACTH_1–24_ nearly 1,000 fold (Dores et al., 2020). Since the R15K and I20 V substitutions in chicken and human ACTH occur in/near the KKRRP motif (Figure 2A), it might lead to different regulatory effects of MRAP1 on MC5R in chicken, although more in-depth studies are still needed to verify this. Furthermore, recent reports have shown that co-expression of MC5R orthologs with MRAP1 in whale sharks, elephant sharks, stingrays, and rainbow trout could increase sensitivity to ACTH_1-24_ (Dores et al., 2018; Barney et al., 2019; Dores et al., 2020; Hoglin et al., 2022), implying that MRAP1’s effect on MC5R sensitivity may be species specific.

In previous work (Zhang et al., 2017), we focused on the expression patterns of *MC3R*, *MC4R*, *MRAP1* and *MRAP2* among the chicken melanocortin system. RNA-Seq and RT-qPCR were used in this work to assess the expression pattern of *MC5R* in Lohmann Layer strain. As illustrated in Figure 4, the highest levels of chicken *MC5R* mRNA were detected in the liver, lung, and adrenal gland. *MRAP2* mRNA was detected mainly in the chicken brain, retina, pituitary, and adrenal gland. The expression of *MRAP1* was limited to the adrenal gland and liver. Our finding was consistent with earlier findings in chicken (Ren et al., 2017; Zhang et al., 2017; Thomas et al., 2018), mouse (Asai et al., 2013) and zebrafish (Sebag et al., 2013). In addition, it also demonstrated that only *MC5R* could exist in liver tissue, which is supported by the RNA-Seq data atlas (Zhang et al., 2022). *MC5R* and *MRAP1* could also be detected in the liver at different developmental stages, especially with the highest expression levels simultaneously after hatch, suggesting that both may have potential physiological effects on the liver metabolism of newborn chicks.

In this study, we found that chicken *MC5R* expression is likely controlled by a functional promoter near exon 1 (within -828 to +171), which contains putative binding sites for many transcriptional factors (such as CREB1, FOXO4, AR, and FOXA1) and displays a strong promoter activity in DF-1 cells. Notably, the promoter activity of P2 (-828/+171) was the highest, 89-fold higher than that of the control. Like its mammalian counterparts, numerous putative transcription factor binding sites were found in the upstream of the *MC5R* transcription start site. According to reports, Foxo4 may bind to the *MC5R* promotor and inhibit *MC5R* transcription in mouse adipocytes (Liu et al., 2017). In the chicken *MC5R* promoter region, a putative FOXO4 binding site was discovered, suggesting that the transcription factor FOXO4 may control *MC5R* transcription. We also found a potential CREB-binding site in the proximal promoter region of the *MC5R* promoter, demonstrating that CREB may be involved in the transcriptional control of *MC5R*. Glucagon has been shown to enhance the intracellular cAMP accumulation and activate CREB (Herzig et al., 2001; Noriega et al., 2011), and the glucagon receptor (GCGR) was highly expressed in the liver of chickens (Wang et al., 2008). In this work, we found that forskolin, 8-Br-cAMP and glucagon enhance the expression of *MC5R* in cultured hepatocytes in a dose-dependent manner. These findings provided support for a role of cAMP/PKA pathway in mediating the increase of *MC5R* mRNA abundance. The present findings established a foundation for further investigation of the regulatory mechanism governing *MC5R* expression in chicken liver tissues. However, further studies are needed to determine the extent that ACTH (or other MSHs) interact with other hormones, such as the fasting-related hormone glucagon, influence liver tissue metabolism.

The high expression of both *MRAP1* and *MC5R* in the liver suggested that the melanocortin ligands (ACTH and other MSHs) may regulate essential physiological functions in this organ. Due to the avian pituitary lacking the intermediate lobe, it secretes relatively little α-MSH, hence ACTH_1–39_ is considered as the primary circulating melanocortin peptide released from pituitary in chickens (Hayashi et al., 1991; Takahashi and Mizusawa, 2013). The ACTH in the plasma was about 10 pg/ml (∼2.2 pmol/l) in 4–6 days chicks determined by a two-site sequential chemiluminescent immunometric assay (Gaston et al., 2017). The concentration of plasma ACTH was about 12 pg/ml in hatched chicks (Okur et al., 2022) and 7–18 pg/ml in three-week-old male chicks from our recent study (Liu et al., 2022). There is little information on the circulating concentrations of chicken α-MSH, which is also known to be present in systemic circulation for regulating pigmentation (Ling et al., 2003) or lipolytic activity (Shipp et al., 2017) in different chicken tissues. The concentration of plasma α-MSH was about 3.1 ng/ml (1.86 nmol/l) in 4-day-old chicks (Shipp et al., 2017). It is unclear if circulating α-MSH is three orders of magnitude higher than circulating ACTH or if this is an artifact of limited investigations. Therefore, we first investigated the effects of ACTH on hepatocytes here. In future research, we intend to reveal the concentration and effects of α-MSH in chickens.

Early studies have tested the effect of continuous ACTH administration in 7-day chickens (Puvadolpirod and Thaxton, 2000a; Puvadolpirod and Thaxton, 2000b; Puvadolpirod and Thaxton, 2000c; Puvadolpirod and Thaxton, 2000d; Thaxton and Puvadolpirod, 2000). Continuous ACTH administration through mini-osmotic pumps offered an ideal model for examining chicken stress, and it was shown that ACTH induced not only an increase in plasma glucose and relative liver weight in this model, but also a fast rise in plasma corticosterone (Puvadolpirod and Thaxton, 2000a). A series of effects of ACTH in the liver were attributed to the release of corticosterone in the adrenal cortex. Corticosterone could mobilize glucose reserves and promote gluconeogenesis *via* fatty acid and protein degradation (Lin et al., 2004). The high expression of MC5R in the liver raises new questions as to whether ACTH can act directly on MC5R/MRAP1 complex to involve in glycolipid metabolism in the liver.

**FIGURE 9:**
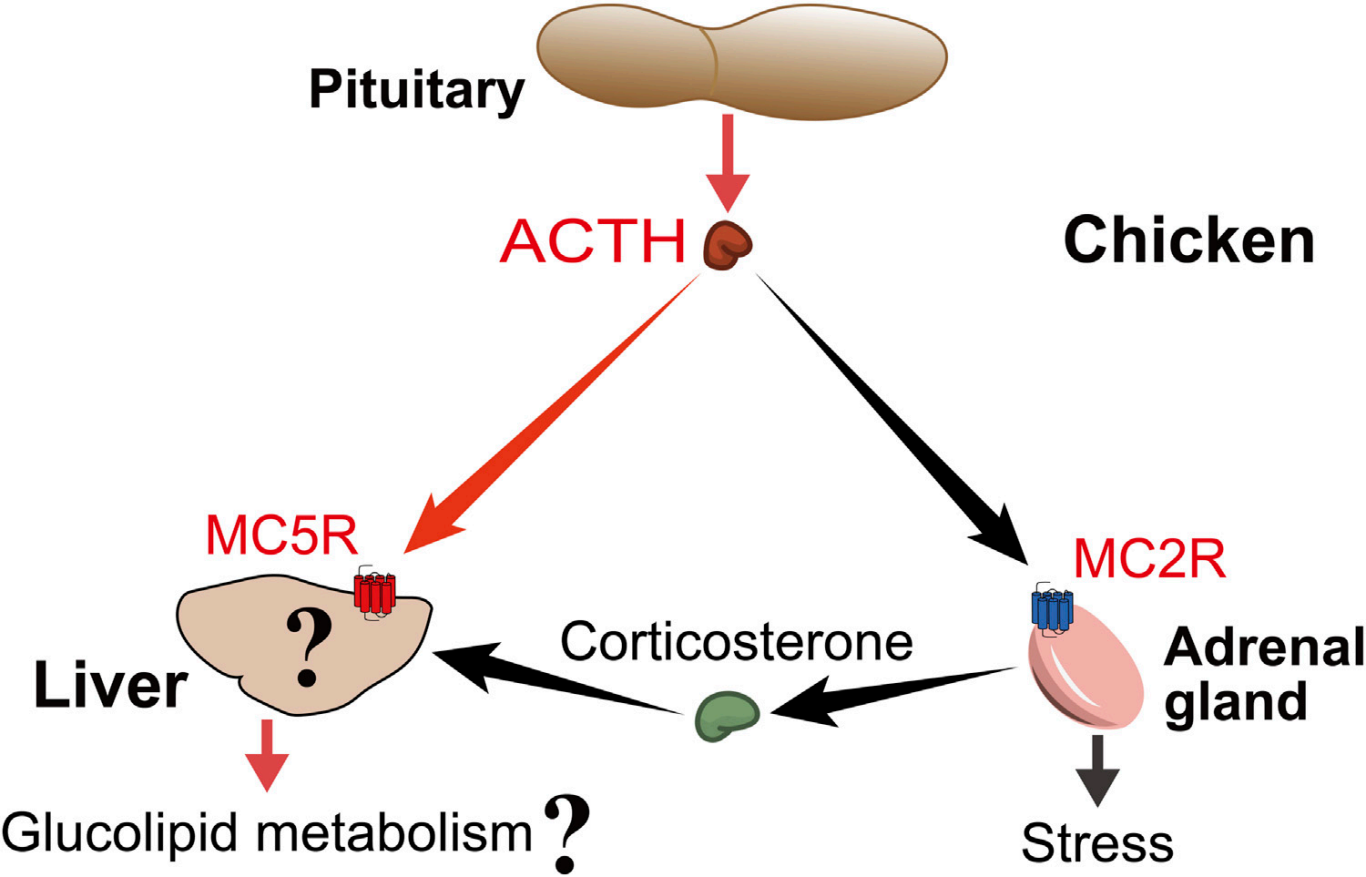
Proposed model for ACTH actions on chicken liver. Pituitary-derived ACTH hormone can act not only on MC2R, which is highly expressed in adrenal tissue, to participate in the stress, but also on MC5R, which is highly expressed in liver tissue, to participate in the regulation of glucolipid metabolism. At the same time, the liver is also the main target organ of ACTH-induced corticosterone, so it is worthwhile to investigate the difference between the direct effect of ACTH and the effect of glucocorticoids.

The present study provided evidence that chicken ACTH can act on primary hepatocytes to increase glucose production and decrease triglyceride contents, while downregulating several genes associated with lipogenesis. Importantly, this result was obtained with physiological concentrations of ACTH. MC5R was implicated in metabolic regulation as it regulated α-MSH signaling in skeletal muscle, which derived glucose disposal and thermogenesis (Enriori et al., 2016). In addition, central activation of MCRs has also been shown to play a role in skeletal muscle glucose uptake in mammals (Nogueiras et al., 2007; Toda et al., 2009). Here, this interesting finding led us to hypothesize that ACTH may directly act on MC5R/MRAP1 complex in chicken hepatocytes to regulate glucose and lipid metabolism, which needs further verification (Figure 9).

The phenomenon of lower *ELOVL6* and *THRSPA* mRNA, instead of *ACACA*, *FASN* and *SCD* mRNA, in the ACTH group has also attracted our attention. All these genes played an important role in hepatic *de novo* lipogenesis (Nematbakhsh et al., 2021). The elongation of very long chain fatty acids protein 6 (ELOVL6) is responsible for the final step in endogenous saturated fatty acid synthesis and involves in *de novo* lipogenesis (Shimano, 2012). The regulation of ELOVL6 expression is important for altering the hepatic lipid composition in response to alterations in dietary and hormonal status (Wang et al., 2006). Thyroid hormone-responsive Spot 14 protein *α* (THRSPA), as a primary lipogenic transcription factor, has emerged as the highest-expressed hepatic gene supporting enhanced lipogenesis and thermogenesis in newly hatched chicks (Resnyk et al., 2013; Cogburn et al., 2018). THRSPA appeared to be a key transcriptional regulator of the switch in metabolism from ectothermy to endothermy (Cogburn et al., 2018). The chicken *THRSPA* gene variations were significantly associated with fat deposition and plasma lipid profiles such as TC and LDL (Hirwa et al., 2010). We demonstrated that ACTH regulates lipogenesis by influencing the expression of *ELOVL6* and *THRSPA* in chicken liver.

Unexpectedly, we found that both forskolin and 8-Br-cAMP did not affect the expression of *ELOVL6* and *THRSPA* genes. In addition to the cAMP pathway *via* Gαs, Activated MC5R can be coupled to the Ca^2+^ pathway *via* Gαq in mammals (Hoogduijn et al., 2002). Moreover, MC5R also can activate some cAMP- and Ca^2+^-independent pathways. For example, MC5R triggers the PI3K-ERK1/2 pathway, which can further mediate downstream pathways in fatty acid re-esterification, cellular proliferation, and immune responses (Rodrigues et al., 2009; Rodrigues et al., 2013; Xu et al., 2022). Then, further investigation will be required to elucidate the exact mechanisms and pathways by which ACTH affects the expression or activity of other transcription factors or downstream metabolic genes in chicken.

In conclusion, we cloned the full-length sequence of chicken *MC5R* gene and characterized its promoter activity in DF1 chicken embryonic fibroblasts. The functional assay demonstrated that MC5R exhibited higher sensitivity to chicken ACTH/α-MSH compared to β-MSH/γ-MSH. It also showed that both MRAP1 and MRAP2 inhibited the trafficking of chicken MC5R to the plasma membrane, and that only MRAP2 significantly reduced the sensitivity of MC5R to stimulation by α-MSH. *MC5R* and *MRAP1* mRNA were co-expressed in the liver of post-hatch chickens. We found that ACTH may increase glucose production, decrease triglyceride content, and dose-dependently downregulate the expression levels of *ELOVL6* and *THRSPA* genes in hepatocytes, suggesting that ACTH has a unique endocrine role in regulating hepatic glucolipid metabolism. However, further studies are needed to characterize and confirm the critical role of the ACTH-MC5R/MRAP1 axis in regulating glucose and lipid metabolism in chicken liver.

## Data Availability

The datasets presented in this study can be found in online repositories. The names of the repository/repositories and accession number(s) can be found below: Nucleotide sequence of chicken MC5R is available in the GenBank databases under the accession number OP259502. The raw data that support the tissue gene expression atlas (TGEA) (https://chickenatlas.avianscu.com/) from adult Lohmann White domestic chickens have been deposited into CNGB Sequence Archive (CNSA) of China National GeneBank DataBase (CNGBdb) with accession number CNP0003404. RNA-Seq data publicly available used to evaluate the expression level of *MC5R* and *MRAP1* at different development stages was downloaded from NCBI under accession code PRJEB26695.
