# Supplementary material for: Characterization of the chicken melanocortin 5 receptor and its potential role in regulating hepatic glucolipid metabolism
Source: Front Physiol. 2022 Oct 6;13:917712. doi: 10.3389/fphys.2022.917712 (PMC9583845; doi:10.3389/fphys.2022.917712)
Supplement: Supplementary file 1 [file Table1.DOCX]

**Supplementary Information**

**Title:**

**Characterization of the Chicken Melanocortin 5 Receptor and its Potential Role in Regulating Hepatic Glucolipid Metabolism**

**Authors:**

Xiao Zhang, Jiancheng Su, Tianjiao Huang, Xinglong Wang, Chenlei Wu, Jing Li, Juan Li, Jiannan Zhang, Yajun Wang

**Supplementary Table 1.** Primers used in this study^a^

| **Gene** | **Sense/antisense** | **Primer sequence (5’-3’)** |
| --- | --- | --- |
| *Primers for cloning of chicken MC5R coding sequence* | | |
| *MC5R* | Sense | gccgaattcCCAGACATGAACACATCCCTCGCAA |
|  | Antisense | gccgaattcTCCTAATATTTGTTTGATAACCCAC |
| *Primers for 5’-RACE* | | |
| *MC5R* | Antisense 1 | CTGTCATGATGTTGTGGTAACGCAGGGCATAG |
|  | Antisense 2 | GTCTACTGCTATTGCCAGCAAACTGCACATGG |
| *Primers for 3’-RACE* | | |
| *MC5R* | Antisense 1 | GCTGCTTTGCCTGGGTACAACTCTGTCCATC |
|  | Antisense 2 | CTATAGCGTGAGAATGGTCTGTGGGTTATC |
| *Primers for amplification of cMC5R promoter regions* | | |
| cMC5R-P1 | Sense | atctgcgatctaagtaagcttCCAAGGGTCTGGCCTGCAG |
|  | Antisense | cagtaccggaatgccaagcttCAGAGCTGCTCCTTCAGCTG |
| cMC5R-P2 | Sense | atctgcgatctaagtaagcttACATTGTATAGCCCCATCAT |
|  | Antisense | cagtaccggaatgccaagcttCAGAGCTGCTCCTTCAGCTG |
| cMC5R-P3 | Sense | atctgcgatctaagtaagcttGCAAGTATTTGCAAATGCAG |
|  | Antisense | cagtaccggaatgccaagcttCAGAGCTGCTCCTTCAGCTG |
| cMC5R-P4 | Sense | atctgcgatctaagtaagcttCACAGTTTGAGTACTGAAGTC |
|  | Antisense | cagtaccggaatgccaagcttCAGAGCTGCTCCTTCAGCTG |
| *Primers for construction of chicken MC5R-HiBiT* | | |
| cMC5R-HiBiT | Sense | TGGCTCGAGCGGTGGCCGAATTCTATGAACA |
|  | Antisense | CTGTGCTGGATATCTGCAGAATTCTCAATATT |
| *Primers for qRT-PCR* | | |
| *THRSPA* | Sense | GTTCTGACCGACCTCACCAA |
|  | Antisense | GTGGGACTTGGCACAGGAAT |
| *ACACA* | Sense | CGTGGTGGAGTTCCAGTTCA |
|  | Antisense | AGAGACCATCCCTCCCATCC |
| *MC5R* | Sense | GGAAGGGTTGCAGGAGATGAAG |
|  | Antisense | GGCTTACAATGCCCAGGGTTAG |
| *ELOVL6* | Sense | CCAGTGGATGCAGGAGAAC |
|  | Antisense | ACAGCAAGGCTCAGGGAC |
| *SCD* | Sense | GGCTGACAAAGTGGTGATG |
|  | Antisense | AGGATGGCTGGAATGAAG |
| *FASN* | Sense | AGCGTGCTATGCTTGCC |
|  | Antisense | GTCCGTGACGAATTGCTTTAT |
| β-actin | Sense | CCCAGACATCAGGGTGTGATG |
|  | Antisense | GTTGGTGACAATACCGTGTTCAAT |

**^a^All primers were synthesized by BGI (China).**
